# Discovery of Four New *FGF5* Variants Causing Long Hair in the Dog

**DOI:** 10.3390/ani16050699

**Published:** 2026-02-24

**Authors:** Robin E. Everts, Tim Roane, Rachael Caron, Cameron Kunstadt, Gabriel Foster, Christa Lafayette

**Affiliations:** AlphaDogDNA by Etalon Inc., Menlo Park, CA 94025, USArachaelcaron25@gmail.com (R.C.);

**Keywords:** dogs, *FGF5*, fibroblast growth factor, genes, hair follicle, missense, recessive, single nucleotide polymorphism

## Abstract

The long hair phenotype of the dog is ascribed to variants in the fibroblast growth factor 5 (*FGF5*) gene. Currently, there are five variant alleles known, Lh1 through Lh5, with any combination of these alleles resulting in a long-haired phenotype in dogs. Recently, genotyping of several long-haired dogs for the known Lh1–Lh5 variants yielded unexpected results as these dogs contained no or only one variant in the *FGF5* gene known to result in the long-hair phenotype. Analysis of the coding region for the *FGF5* gene identified four novel variants associated with the long-haired phenotype. One dog of mixed ancestry had an insertion of G very similar to and near to the long-hair variant Lh4, NC_006614.4: g.37352832insCC. A group of 24 Tibetan Mastiffs showed the Lh1 variant NC_006614.4:g.37372096C>A in 11 dogs only, and no other known Lh variant. Within the coding frame of the *FGF5* gene, three unknown variants were discovered that were predicted to have a phenotypic effect. These new variants were inherited in a Mendelian fashion and, in combination with one of the known *FGF5* alleles, likely predict the long-hair phenotype. Lastly, eight of the 24 Tibetan Mastiff dogs carried three *FGF5* variants.

## Simple Summary

The long-hair phenotype in dogs is a recessive trait and is caused by five known variants in the *FGF5* gene. During a standard genotyping procedure, twenty-two Tibetan Mastiffs and one mixed-breed dog were classified as short-haired, even though they phenotypically clearly showed a long hair phenotype. Re-analysis of their genotype data showed these dogs did not have two known long hair variants. However, it was discovered that these dogs contained other variants in the coding sequence of *FGF5*. The new variants were inherited in a Mendelian fashion, and dogs with only two putative new variants exhibited the long-hair phenotype, showing these new alleles, together with known variants, can predict the long-hair phenotype.

## 1. Introduction

Dogs show tremendous variation between breeds, as a result of careful selection over time and rigorous breed standards. As has been demonstrated for coat color [1] and coat length [2], simple genetics explains most of the variation seen. For dog breeds, there are three main coat variations that are factored into breed standards: coat color, hair length, and hair type. Because breed standards have specific hair length, incorrect hair length can be a reason for excluding dogs from breed programs.

It was discovered in 2006 that hair length in dogs is due to variations in the *FGF5* gene [2]. The wild-type variant of the *FGF5* gene produces short hair, and any combination of the known mutations in the *FGF5* gene results in dogs with a long hair phenotype. In other species such as cats, donkeys, and humans, *FGF5* has also been found to play a crucial role in hair length [3,4,5]. To date, in dogs, five different variants have been discovered, named Lh1 to Lh5 (see https://omia.org/OMIA000439/9615/ (accessed on 5 January 2026 [6])). Variant Lh1 is found in exon 1, Lh2, Lh3, and Lh4 are nucleotide changes in exon 3, and Lh5 is a variant in intron 1 [7]. For more details on these variants, see Table 1.

These five *FGF5* variants (Lh1–Lh5) have been used to predict the hair length phenotype in dogs, but recent Tibetan Mastiff cases showed discrepancies between the observed hair phenotype and the results of the standard Lh1–Lh5 genotyping. As Tibetan Mastiffs are known for their thick and dense coats with medium to long hair, a retrospective analysis of data from the *FGF5* gene exon regions was performed to discover whether other DNA variants were present that could account for this discrepancy.

## 2. Materials and Methods

### 2.1. Animals

Twenty-four Tibetan Mastiff dogs and one mixed breed dog were sent in for comprehensive genetic testing. The respective owners notified us of a discrepancy between the long hair phenotype observed for most of the dogs and their reports specifying the dogs as short-haired. Review of the data for the dogs in question showed mostly wild-type genotype calls for *FGF5* variants Lh1–Lh5. Of these dogs, twenty-two of the Tibetan Mastiff dogs and the mixed breed dog showed hair length longer than that predicted by their *FGF5* genotype and were selected for an in-depth analysis of the *FGF5* exonic regions after obtaining owner consent. Hair length for a subset of these 23 dogs was measured (in inches) at the shoulders using a ruler. In addition, genotype data for the known and new *FGF5* variants for another 714 dogs were also collected for comparison.

### 2.2. DNA Extraction and Sequencing

Genomic DNA was extracted from buccal-swab samples (DNA Genotek Inc., Stittsville, ON, Canada) using the Puregene Extraction Kit following the manufacturer’s protocol (QIAGEN, Inc., Germantown, MD, USA). DNA sequencing libraries were prepared using the manufacturer’s protocols from 150 ng of genomic DNA using the Twist Library Prep Kit EF 2.0 kit with the Twist Universal Adapter kit (Twist Bioscience, San Francisco, CA, USA). Subsequently, an overnight hybridization capture reaction, with 16 samples per pool, was performed with the Standard Hybridization Reagent Kit (Twist Bioscience, San Francisco, CA, USA) using the manufacturer’s protocol with the addition of canine Hybloc DNA (Applied Genetics, Melbourne, FL, USA). Sequencing was performed on a P2 flow cell using 2 × 150 bp sequencing on a NextSeq1000 instrument (Illumina, San Diego, CA, USA) as per the manufacturer’s protocol. Probe sequences for the *FGF5* exons in which the known and novel variants are located are provided in Supplemental Table S1.

### 2.3. Data Analysis

All sequence reads were trimmed of adapter sequences using cutadapt. The resulting sequences had base call quality scores ≥ Q30 and read depth > 40× for all regions under investigation after alignment to canFam6.0 (Dog10K_Boxer/Tasha) [8] using BWA-MEM2 [9]. After alignment to the genome, the data were processed using an in-house variant caller pipeline based on samtools [10], and the genotype results for the coat, color, health, parentage, and ancestry were reported out in vcf format. For this current report, Integrative Genomics Viewer (IGV) version 2.16.2 [11] was then used to review the resulting BAM files for the presence of the newly identified variants in the *FGF5* coding region on chromosome 32. All genome positions in this communication are based on canFam6.0 unless otherwise noted. Novel variants were analyzed for predicted significance using PolyPhen-2 (http://genetics.bwh.harvard.edu/pph2/ (accessed on 5 January 2026)) and SIFT (https://sift.bii.a-star.edu.sg/ (accessed on 5 January 2026)).

For a global analysis, data for the known (Lh1–Lh5) and new long hair alleles (Lh6–Lh9) were extracted from vcf files for another 714 previously genotyped animals and used to assess allele frequencies and breed specificities.

## 3. Results

For the twenty-three dogs with a discrepancy between the actual and predicted phenotype based on their *FGF5* genotype, we discovered four putative new functional variants in the coding region of the *FGF5* gene. To ascertain whether these variants were present in other dogs, we extracted the genotypes for these nine variants in total from the respective BAM files for 714 additional dogs (Supplemental Table S3). All variants were covered by a minimum of 40x read depth for these samples. The Lh1 allele was the most frequent with an allele frequency of 30%, while Lh7, Lh8, and Lh9 were distant second with approx. 1% frequency. The latter could be biased due to the fact that this study contained many Tibetan Mastiffs. Of the putatively new variants, only Lh7 was seen in another breed, the Anatolian shepherd.

### 3.1. Putative Lh6 Variant in a Dog of Unknown Heritage

**Figure 1:**
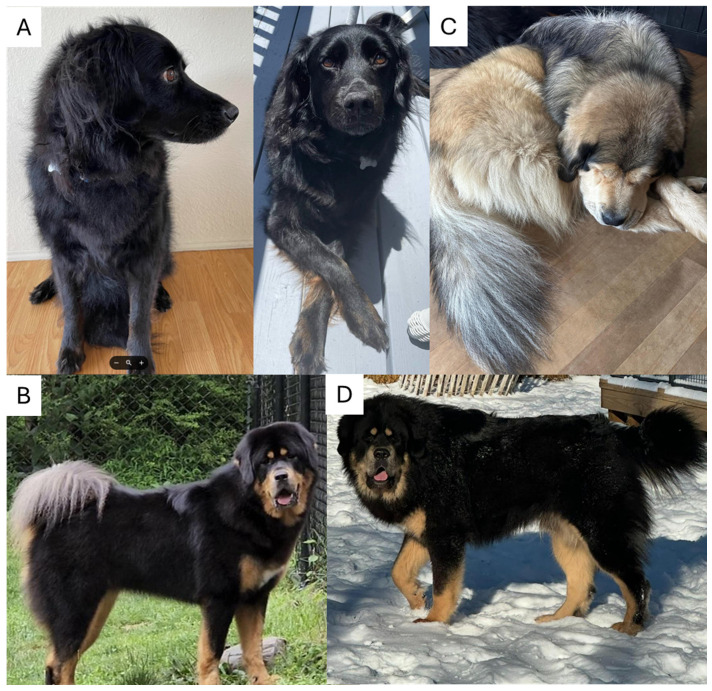
(**A**) dog of unknown breed with a new long hair variant Lh6 in addition to Lh2. (**B**) Example of the Lh7/Lh9 heterozygous phenotype. (**C**) Example of the Lh8/Lh8 homozygous phenotype. (**D**) Example of the Lh9/Lh9 homozygous phenotype.

**Figure 2:**
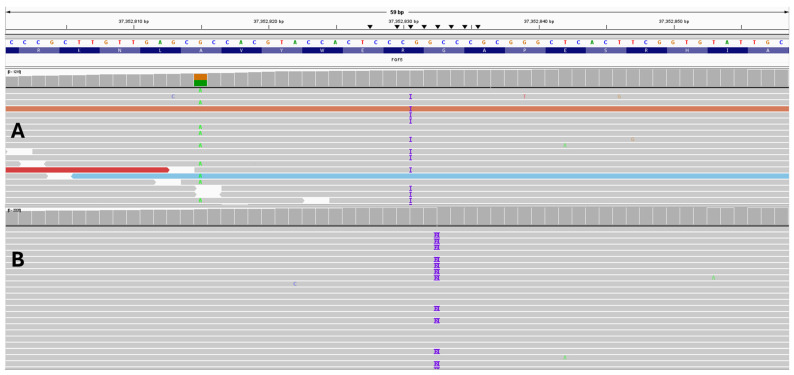
Example of the insertion of a single G at NC_006614.4:g.37352830-37352831insG (**A**), and it is very close to the CC insertion of Lh3 at NC_006614.4:g.37352832-37352833 as shown for a French Bulldog right below (**B**). In (**A**), to the left, the dog’s other mutation NC_006614.4:g.37352815G>A (Lh2) is clearly shown. The sequence reads also clearly show these two variants are on different sequence strands; therefore, they are on different parental alleles. This new variant, NC_006614.4:g.37352830-37352831G>GG, is putatively called Lh6. Both samples had read depths of >1000x for the region under investigation.

A dog with a mixed ancestry of Poodle, Rottweiler, German Shepherd, and Cocker Spaniel (See Supplemental Figure S1) was genotyped and showed only a single *FGF5* long-hair allele (Lh2/Sh). Under a recessive model, this genotype should not produce long hair; nevertheless, the dog possessed long hair with hair length at the shoulders and breaches measuring >2 inches. (See Figure 1A). Analysis of the raw bam files using IGV showed this dog had a single copy of the Lh2 variant, NC_006614.4:g.37352815G>A, and a previously unknown variant just in front of β sheet 9 of the *FGF5* protein, with an insertion of a G nucleotide at NC_006614.4:g.37352830-37352831insG; NM_001048129:c.562_563insC; NP_001041594.1:p.(R188fxX12) (Figure 2 and Figure S2). This variant causes a frameshift change. Based on the insertion of a C nucleotide in the coding sequence, this variant is tentatively assigned p.R188PfsX12. This C insertion variant in the coding sequence is in very close proximity and similar to the Lh4 variant, which is an insertion of CC in the coding sequence, at position NC_006614.4:g.37352832-37352833insGG, and is also located within the 16 bp deletion of the Lh3 variant. We tentatively call this G insertion variant Lh6. This variant was not seen in any of the other 738 dogs analyzed.

### 3.2. Tibetan Mastiff

Of the twenty-four Tibetan Mastiff dogs tested for known *FGF5* variants, two dogs were homozygous Lh1/Lh1 genotype and thus had a long-haired phenotype. For the other 22 dogs, the genotype results, based on known alleles of the *FGF5* gene, would be indicative of a short-haired phenotype for their coat length as they either had a single known variant (*n* = 9) or no known variant at all (*n* = 13). However, the hair length was measured at a minimum of 5 inches at the shoulder (Supplemental Figure S3), and the shawl (i.e., manes around the neck) exceeded 7 inches in length. Because Tibetan Mastiffs are phenotypically long-haired (as per AKC standards), review of the bam files for the region containing the *FGF5* gene was performed, and three new putative long hair variants were discovered.

### 3.3. Putative Lh7 Variant

**Figure 3:**
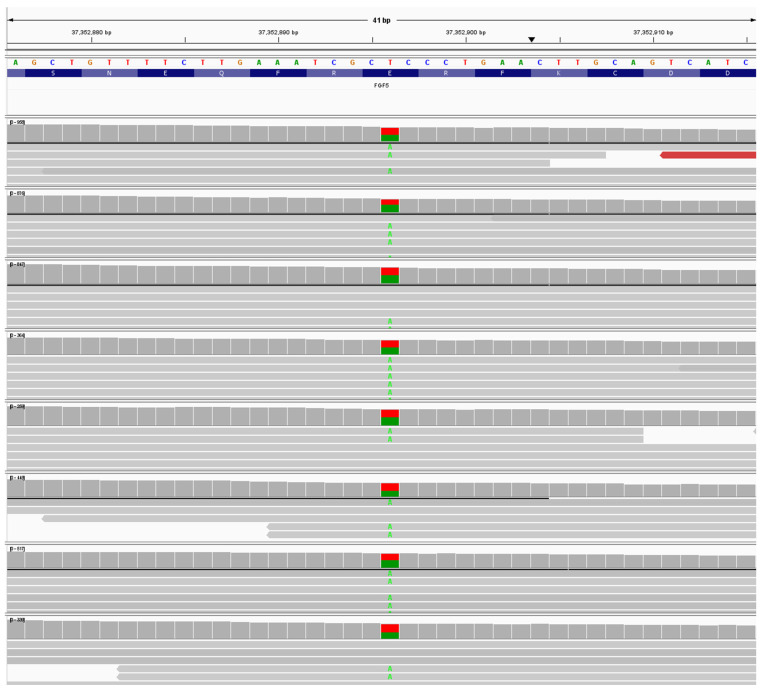
Example of an IGV window showing seven Tibetan Mastiff dogs with a heterozygous call for the variant NC_006614.4:g.37352896T>A. This variant is putatively called Lh7. Samples had min. read depth of 300x for the region shown.

There were 13 dogs heterozygous for NC_006614.4:g.37352896T>A; NM_001048129:c.497A>T NP_001041594.1:p.(E166V). See Figure 3. The variant NC_006614.4:g.37352896 is a T>A variant in exon 3, causing a Glutamic acid to Valine substitution in amino acid 166 (See Supplemental Figure S4) in β sheet 7 of the *FGF5* protein. Substitution of glutamic acid with valine likely results in a significant change in the protein structure, as glutamic acid is hydrophilic, whereas valine is a hydrophobic amino acid. Analyzing the variant NC_006614.4:g.37352896T>A by PolyPhen-2 (score 0.999) and SIFT analysis (score 0.000) showed this variant as “Probably damaging”. This variant, tentatively called Lh7, was also seen in an Anatolian Shepherd. An example of an Lh7/Lh9 dog is shown in Figure 1B.

### 3.4. Putative Lh8 Variant

**Figure 4:**
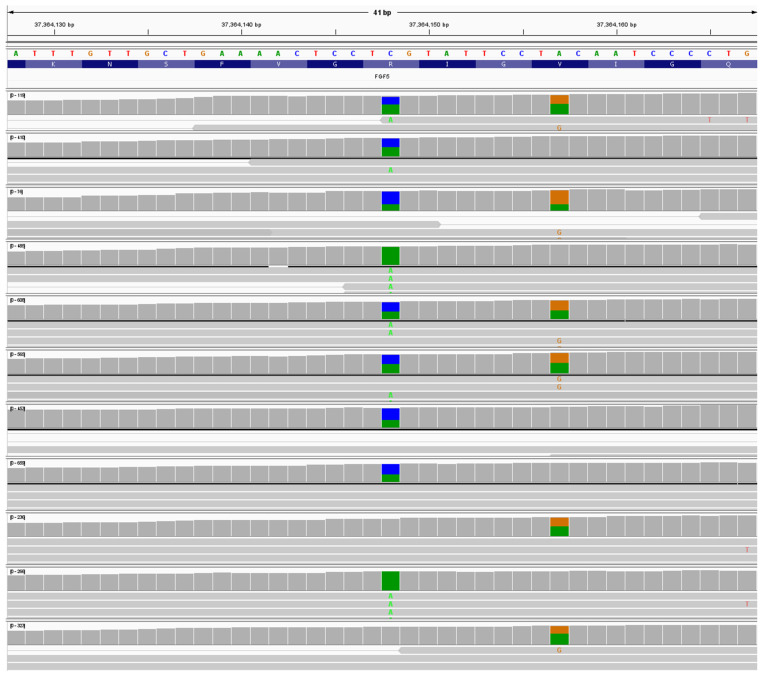
Example of IGV window for nine Tibetan Mastiff dogs with heterozygous and homozygous animals for the variants Lh8: NC_006614.4:g.37364148C>A (blue/green) and Lh9: NC_006614.4:g.37364157A>G (orange/green). The samples had min. read depth of 200x for the region shown. As shown above, the variants Lh8 and Lh9 are not in-phase and occur on alternate alleles of the *FGF5* gene.

There were 13 heterozygous dogs, and two homozygous dogs for NC_006614.4:g.37364148C>A; NM_001048129:c.410G>T; NP_001041594.1:p.(R137L). See Figure 4. The variant NC_006614.4:g.37364148 is a Cytosine to Adenosine variant causing an Arginine to Leucine substitution in amino acid 136 (See Supplemental Figure S5) in β sheet 4 of the *FGF5* protein. Analysis of the resulting protein with PolyPhen-2 (score 0.991) and SIFT analysis (score 0.001) showed this variant as “Probably damaging”. This variant is tentatively called Lh8 and was not seen in any of the other 715 dogs analyzed. An example of an Lh8/Lh8 dog is shown in Figure 1C.

### 3.5. Putative Lh9

Lastly, 11 dogs were heterozygous, and one dog was homozygous for NC_006614.4:g.37364157A>G; NM_001048129:c.398T>C; NP_001041594.1:p.(V133A). See Figure 4. The variant NC_006614.4: g.37364157 is an A>G variant causing a Valine to Alanine substitution in amino acid 133 (V133A) (See Supplemental Figure S6) in β sheet four of the *FGF5* protein. PolyPhen-2 (score 1.000) and SIFT analysis (score 0.000) showed this variant as “probably damaging”. This variant is tentatively called Lh9 and was not seen in any of the other 715 dogs analyzed. An example of an Lh9/Lh9 dog is shown in Figure 1D.

The majority of the dogs had only two variants present, either a known variant from Lh1 through Lh5, and a new variant from Lh6 through Lh9, or only new variant alleles Lh6 through Lh9. As the *FGF5* variants are known to be recessive, i.e., only cause long hair if at least two variants are present, it is highly likely these new variants are causative variants, as evidenced by thirteen of the 24 long-haired Tibetan Mastiffs showing only a combination of the new putative alleles Lh6 through Lh9.

**Figure 5:**
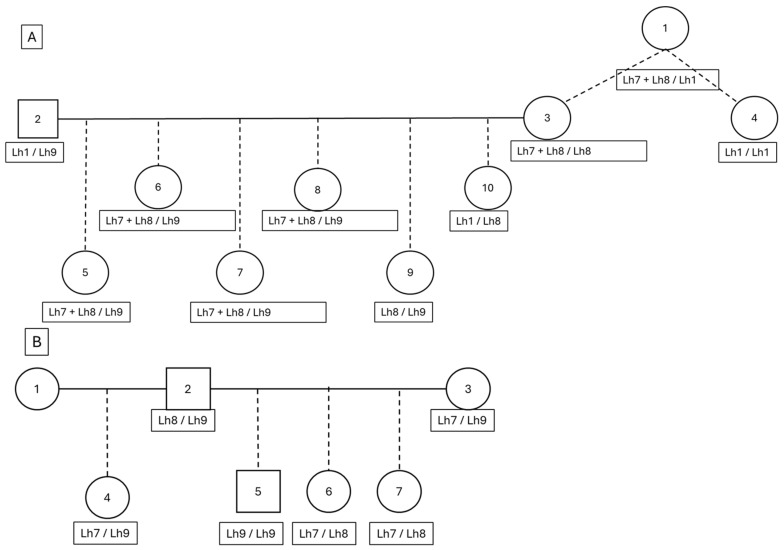
Examples of two families of phenotypically long-haired Tibetan Mastiffs showing the respective putative long hair variants Lh7, Lh8, and Lh9 in this breed. (**A**) A family with the four variants Lh1, Lh7, Lh8, and Lh9 is present, of which several dogs show three variants. The variants Lh7 and Lh8 are shown to co-occur on the same allele of the *FGF5* gene. (**B**) A family with three variants, Lh7, Lh8, and Lh9, is present with two variants per dog. Females shown as circles, males as squares. As all dogs show the long-hair phenotype, variants Lh7 and Lh8 are putative functional variants causing the long-haired phenotype.

Additionally, eight dogs presented three *FGF5* variants in their genotype results. As can be seen in Figure 5, there were families where either three (A) or two (B) variants are segregating in the pedigree. As the pedigree in Figure 5A showed animal 4 to be homozygous Lh1/Lh1, it was deduced that the Lh7 and Lh8 variants in animal 1 were on the same parental chromosome. This was also observed in its offspring (animal 3) and its offspring (animals 5–10). The variants Lh7 and Lh8 were also present without other variants on a parental chromosome, as can be seen in the family in Figure 5B.

## 4. Discussion

Currently, there are five known long hair variants for *FGF5* that have been observed in dog breeds with long hair phenotypes. However, our analysis of 24 long-haired Tibetan Mastiff dogs and one mixed breed dog showed only two dogs homozygous for Lh1, nine heterozygous for Lh1 only, one dog heterozygous for Lh2, and 13 dogs without a known Lh1 through Lh5 allele (Supplemental Table S2). The majority of these dogs (23 of 25 dogs) would be considered short-haired based on the known *FGF5* long hair variants. Owner feedback, pictures of the dogs in question, and measurement of the hair length clearly showed that the animals in question were phenotypically long-haired. Variation in the hair length measured was likely due to sex and age of the dogs, which were not recorded or taken into account. We therefore analyzed the whole *FGF5* exonic region and found three different variants present in Tibetan Mastiff dogs that are all predicted to have a severe impact on the protein structure according to PolyPhen-2 and SIFT (Table 2). In the one dog of mixed breed that was genotyped as Lh2/n, analysis of the *FGF5* coding regions showed the animal harbored an insertion of a G nucleotide in the vicinity of the Lh4 allele, the latter being an insertion of two C nucleotides. The effect of this insertion is a similarly altered protein sequence. See Supplemental Figure S7 for an alignment of all nine variants.

Unexpectedly, eight dogs presented with three variants in the *FGF5* gene. In six dogs, it was deduced from the pedigree information that the Lh7 and Lh8 were on the same parental chromosome, approx. 11 kb apart. The other two dogs, based on pedigree analysis, were related to the six dogs within 2–3 generations, and it was assumed they also carried the Lh7 and Lh8 on the same chromosome. This may present a problem for genotyping, as dogs that carry a Lh7 and Lh8 variant could be carrying both variants on the same chromosome. A similar situation has been observed for the B locus, where the b^c^ and b^d^ variants were found to be on the same chromosome in certain breeds [12].

The four new variants presented here are likely not as prevalent as some of the previously identified alleles, but they will help in providing more accurate phenotype predictions for hair type in certain breeds that have been derived in part from the Tibetan Mastiff. Tibetan Mastiffs are an ancient breed and may have been brought into Europe in the 1300s. They are a founder animal for large breeds such as Anatolian Shepherd, Old English Shepherd, Rottweiler, and Saint Bernard [13], and as such, these new alleles might be present in these breeds too, as was shown for the Anatolian Shepherd (Table 3). All the prior known *FGF5* variants Lh1–Lh5, as well as the newly discovered variants Lh6–Lh9, are located in or near the β sheets of the *FGF5* protein (Supplementary Figure S7). These β sheets are the structures within the FGF5 protein that allow it to fold correctly. Alterations to these β sheets likely inhibit the correct folding and render the protein less effective at its primary function of hair cycle regulation.

## 5. Conclusions

Our study has identified four new variants in the *FGF5* gene in dogs that possessed long hair, but they were reported as short-haired using genotypes composed of known variants, Lh1 through Lh5. Although the long hair variants in the *FGF5* gene are inherited as an autosomal recessive trait, there have been reports of dogs where an incomplete dominant inheritance was expected based on the five known variants. Perhaps screening for these (or other) new variants may reveal that they are missing variants. The new alleles Lh7 through Lh9 have only been found in dogs of Tibetan Mastiff ancestry, and more studies are warranted in larger sample sets to see their real prevalence. In the case of the Lh6 allele in the mixed-breed dog, examining a larger cohort is needed. This dog was of mixed Poodle, Rottweiler, German Shepherd, and Cocker Spaniel ancestry, all breeds known to carry Lh1. Overall, genotyping these new variants in larger dog populations will provide a better understanding of their breed prevalence.

## Figures and Tables

**Table 1:** Existing long hair variants in FGF5 (OMIA ID: 000439).

Variant (OMIA ID)	Position	Amino Acid Change	Protein Change	Breed Found	Reference
Lh1 (48)	NC_006614.4:g.37372096C>A	NM_001048129:c.284G>T	NP_001041594.1:p.C95F	many	[2]
Lh2 (104)	NC_006614.4:g.37352815G>A	NM_001048129:c.578C>T	NP_001041594.1:p.A193V	Akitas, Siberian huskies, Samoyeds	[7]
Lh3 (952)	NC_006614.4:g.37352821-37352836del16	NM_001048129:c.556-571	NP_001041594.1:p.A186Tfs71	Eurasier	[7]
Lh4 (950)	NC_006614.4:g.37352831-37352832dupCC	NM_001048129:c.559-560dup	NP_001041594.1:p.R188Afs75	Afghan hounds, Eurasier	[7]
Lh5 (418)	NC_006614.4:g.37364202A>T	NM_001048129:c.368-11T>A	Prevents splicing of exon 2	Afghan hounds	[7]

**Table 2:** The new long hair variants in *FGF5* (OMIA ID: 000439).

Variant (OMIA ID)	Position	Amino Acid Change	Protein Change	PolyPhen-2 Score	SIFT Score	Breed Found	Reference
Lh6	NC_006614.4: g.37352830-37352831insG	NM_001048129: c.562_563insC	NP_001041594.1: p.R188PfsX12	Not analyzed	0.02	Mixed breed dog	This paper
Lh7	NC_006614.4: g.37352896T>A	NM_001048129: c. 497A>T	NP_001041594.1: p.E166V	0.999—probably damaging	0.00	Tibetan Mastiff	This paper
Lh8	NC_006614.4: g.37364148C>A	NM_001048129: c. 410G>T	NP_001041594.1: p.R137L	0.991—probably damaging	0.01	Tibetan Mastiff	This paper
Lh9	NC_006614.4: g.37364157A>G	NM_001048129: c. 398T>C	NP_001041594.1: p.V133A	1.000—probably damaging	0.00	Tibetan Mastiff	This paper

**Table 3:** All *FGF5* alleles, known and new, and their presence in the dogs in our data set (*n* = 739). Minor Allele Frequency (MAF) is measured over all dogs.

Position on NC_006614.4:	Allele	MAF	Breeds
37,372,096	Lh1	30.08%	43 purebred breeds and multiple mixed breeds: Affenpinscher, Akita, Alaskan Klee Kai, Anatolian Shepherd Dog, Australian Cattle Dog, Australian Shepherd, Basenji, Belgian Malinois, Belgian Sheepdog, Belgian Tervuren, Bernedoodle, Bernese Mountain Dog, Border Collie, Brittany, Bullmastiff, Cavalier King Charles Spaniel, Chihuahua, Cocker Spaniel, Collie, Curly Coated Retriever, Dachshund, English Springer Spaniel, French Bulldog, German Shepherd, Golden Retriever, Great Pyrenees, Labradoodle, Labrador Retriever, Norfolk Terrier, Papillon, Pekingese, Pomsky, Poodle, Poodle (Toy), Portuguese Water Dog, Rottweiler, Russell Terrier, Sheepadoodle, Tibetan Mastiff, Toy Poodle, West Highland White Terrier, Xoloitzcuintli, Yorkshire Terrier
37,352,815	Lh2	0.34%	Siberian Husky, Mixed breeds
37,35,2821	Lh3	0.07%	Australian Cattle Dog
37,352,832	Lh4	0.20%	French Bulldog, Belgian Malinois
37,364,202	Lh5	0.00%	none
37,352,830	Lh6	0.07%	Mixed breed dog (Poodle, German Shepherd, Rottweiler)
37,352,896	Lh7	0.95%	Tibetan Mastiff and Anatolian Shepherd dog
37,364,148	Lh8	1.15%	Tibetan Mastiff
37,364,157	Lh9	0.88%	Tibetan Mastiff

## Data Availability

The variant data for this study have been deposited in the European Variation Archive (EVA) at EMBL-EBI under accession number PRJEB98564.

## Supplementary Materials

The following supporting information can be downloaded at: https://www.mdpi.com/article/10.3390/ani16050699/s1, Supplemental Figure S1: Ancestry outcome of the dog Otter with mixed ancestry; Supplemental Figure S2: Variant Lh6, NC_006614.4:g.37352830-37352831 insertion G; Supplemental Figure S3: Two examples of hair measures; Supplemental Figure S4: Variant Lh7, NC_006614.4:g.37352896 T>A; Supplemental Figure S5: Variant Lh8, NC_006614.4:g.37364148 C>A; Supplemental Figure S6: Variant Lh9, NC_006614.4:g.37364157 A>G; Supplemental Figure S7: Protein sequences of all 9 Long hair variants and their changes; Supplemental Table S1: Hybrid Capture Probe sequences for the 5 known and 4 putative new alleles; Supplemental Table S2: Number of dogs and their respective alleles before and after adding the new variants as presented in this paper; Supplemental Table S3: FGF5 genotype data for all dogs in the study.
