# Supplementary material for: Discovery of Four New FGF5 Variants Causing Long Hair in the Dog
Source: Animals (Basel). 2026 Feb 24;16(5):699. doi: 10.3390/ani16050699 (PMC12983947; doi:10.3390/ani16050699)
Supplement: Supplementary file 1 [file animals-16-00699-s001.zip › Supplemental_file_1a.docx]

Supplemental Figure S1. Ancestry outcome of the dog Otter with mixed ancestry


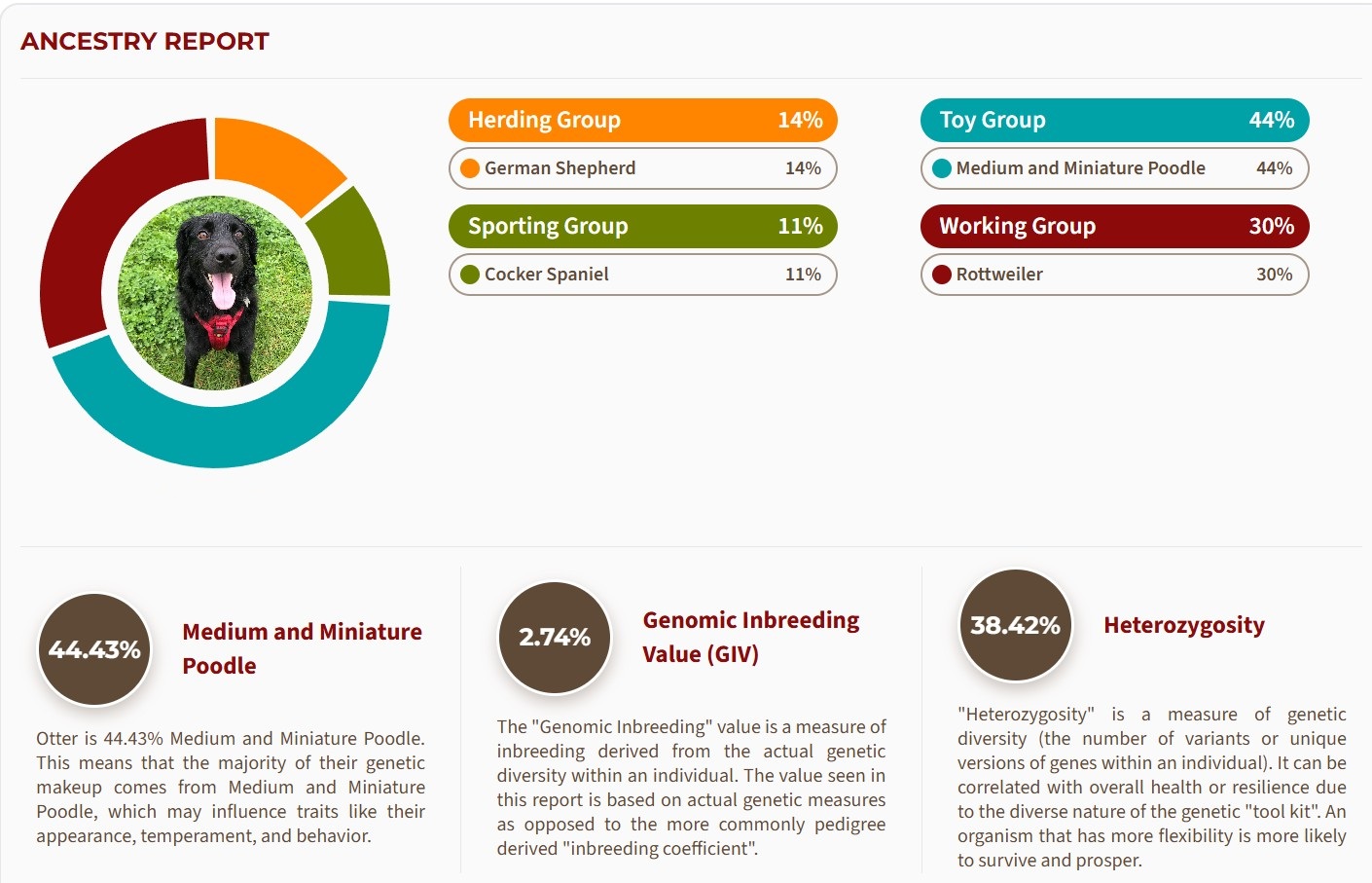


Supplemental Figure S2. Variant Lh6, NC_006614.4:g.37352830-37352831 insertion G. As the gene is reverse complemented on the DNA, the insertion depicted here is a C. It disrupts the reading frame and causes an early termination codon (TAA).

Supplemental Figure S3. Two examples of hair measures. Using a ruler, hair on shoulder/neck is shown to be approx. 5 – 8 inches long for the Tibetan Mastiff.


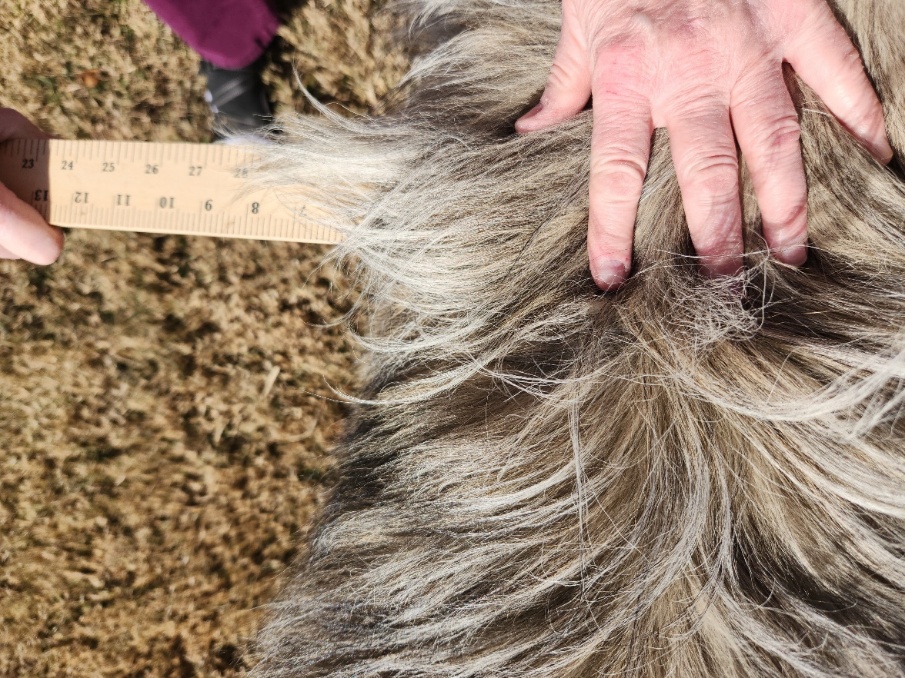

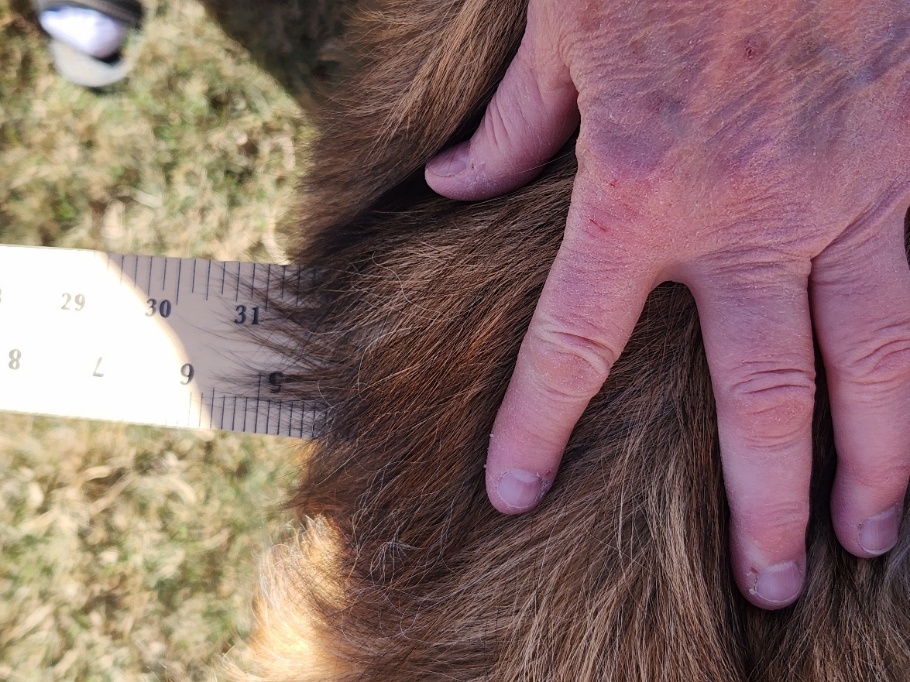


Supplemental Figure S4. Variant Lh7, NC_006614.4:g.37352896 T>A. As the gene is reverse complemented on the DNA, the variant depicted here is a T. This changes a GAG (Glutamic acid) to a GTG (Valine)

Supplemental Figure S5. Variant Lh8, NC_006614.4:g.37364148 C>A. As the gene is reverse complemented on the DNA, the variant depicted here is a T. This changes a CGA (Arginine) to a CTA (Leucine)

Supplemental Figure S6. Variant Lh9, NC_006614.4:g.37364157 A>G. As the gene is reverse complemented on the DNA, the variant depicted here is a C. This changes a GTA (Valine) to a GCA (Alanine)

Supplemental Figure S7. Protein sequences of all 9 Long hair variants and their changes (bold red in yellow background). The beta sheets are indicated as the underlined protein sequence.


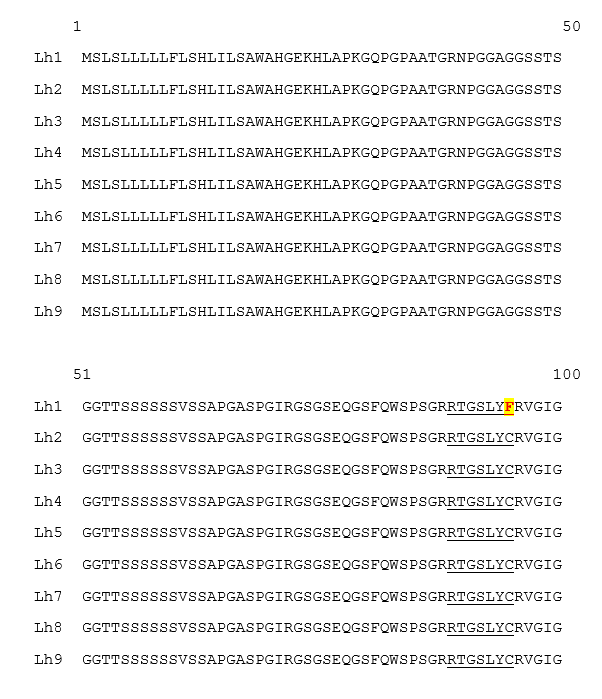


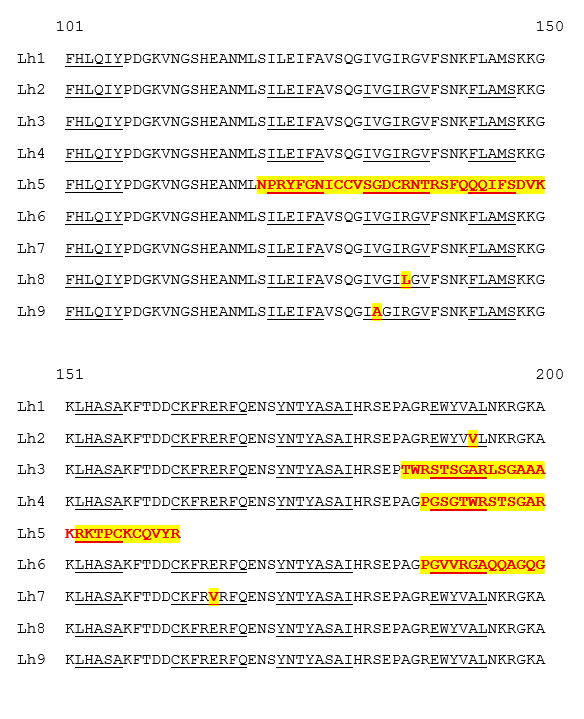


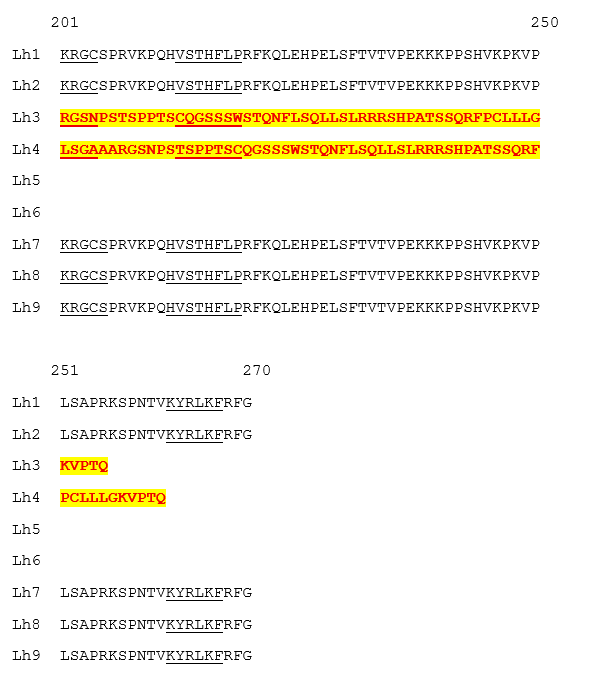


Supplemental Table S1. Hybrid Capture Probe sequences for the 5 known and 4 putative new alleles.

| Variant | Hybrid Capture Probe Sequence (5’=3’) |
| --- | --- |
| Lh1 | ATATTGGCTTCCTGGGAGCCATTGACTTTGCCATCCGGGTAGATCTGCAGATGGAAACCG  ATGCCCACTCTGCAGTAGAGGCTGCCGGTCCGGCGCCCCGAGGGGCTCCACTGGAAGCTG |
| Lh2, Lh3, Lh4 | ATTTCAAGAAAACAGCTATAACACCTACGCCTCCGCAATACACCGAAGTGAGCCCGCGGG  CCGGGAGTGGTACGTGGCGCTCAACAAGCGGGGCAAGGCTAAGCGGGGCTGCAGCCCGAG |
| Lh5 | CTCCTCGTATTCCTACAATCCCCTGAGACACAGCAAATATTTCCAAAATACCTAGGATGA  CAGAAATCCCAAAATAAAACACAGACTCCTCTATAAATCATGGTGAATTCAATTTCTACA |
| Lh6, Lh7 | ATTTCAAGAAAACAGCTATAACACCTACGCCTCCGCAATACACCGAAGTGAGCCCGCGGG  CCGGGAGTGGTACGTGGCGCTCAACAAGCGGGGCAAGGCTAAGCGGGGCTGCAGCCCGAG |
| Lh8, Lh9 | CTCCTCGTATTCCTACAATCCCCTGAGACACAGCAAATATTTCCAAAATACCTAGGATGA  CAGAAATCCCAAAATAAAACACAGACTCCTCTATAAATCATGGTGAATTCAATTTCTACA |

Supplemental Table S2. Number of dogs and their respective alleles before and after adding the new variants as presented in this paper.

| Original FGF5 genotype | Number of dogs | Including new alleles | Number of dogs |
| --- | --- | --- | --- |
| Lh1/Lh1 | 2 | Lh1/Lh1 | 2 |
| Lh1/Sh | 9 | Lh1/Lh7 | 1 |
|  |  | Lh1/Lh8 | 2 |
|  |  | Lh1/Lh7 + Lh8 | 3 |
|  |  | Lh1/Lh9 | 3 |
| Lh2/Sh | 1 | Lh2/Lh6 | 1 |
| Sh/Sh | 13 | Lh7/Lh9 | 2 |
|  |  | Lh7/Lh8 | 2 |
|  |  | Lh8/Lh8 | 1 |
|  |  | Lh8/Lh7 + Lh8 | 1 |
|  |  | Lh8/Lh9 | 2 |
|  |  | Lh9/Lh9 | 1 |
|  |  | Lh9/Lh7 + Lh8 | 4 |
