# Supplementary figures and images for: Discovery of Four New FGF5 Variants Causing Long Hair in the Dog
Source: Animals (Basel). 2026 Feb 24;16(5):699. doi: 10.3390/ani16050699 (PMC12983947; doi:10.3390/ani16050699)

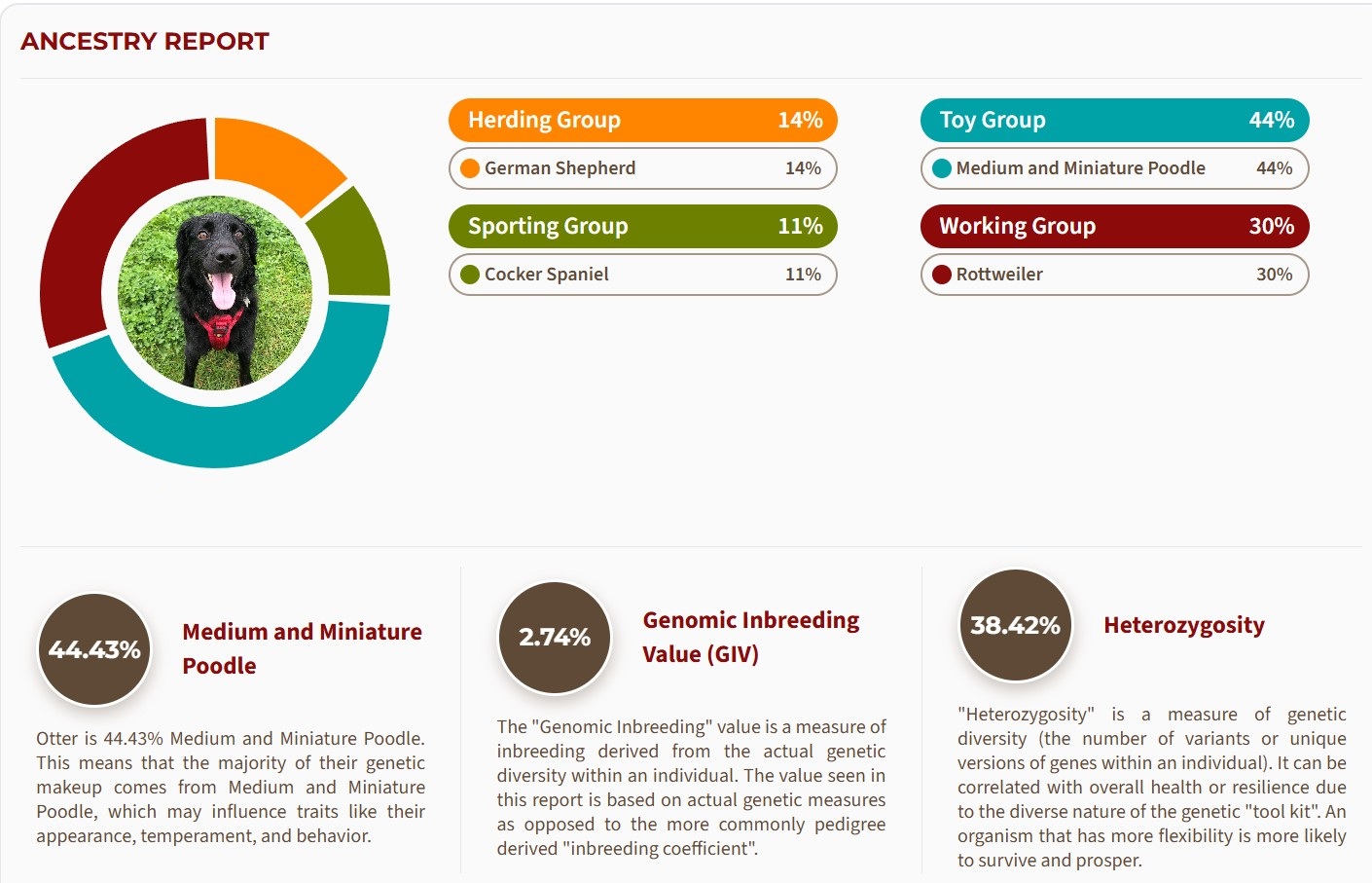

Supplement: Supplementary file 1 [file animals-16-00699-s001.zip › Suppl_Fig1.jpg]
